# A Study of Head Lice Infestation (Pediculosis Capitis) among Primary School Students in the Villages of Abadan in 2012

**Published:** 2014-07

**Authors:** Shayesteh Salehi, Maryam Ban, Minoo Motaghi

**Affiliations:** 1 Department of Nursing, Khorasgan (Isfahan) Branch, Islamic Azad University, Isfahan, Iran

**Keywords:** Head Lice, Infestation, Pediculosis, Primary School

## Abstract

Head lice contamination has a global distribution and it is regarded as a main health problem throughout the world. Given the importance of physical health of students and head lice prevalence at school age, we aimed to examine the rate of head lice contamination among primary school students in the villages of Abadan in 2012. In this descriptive study, 624 students were randomly selected from primary schools. The hair of all students under the study was examined by the researcher (community health nurse) and the result was registered in the checklist confidentially. Moreover, one questionnaire including personal and family information was filled out for each student. The obtained data were later analyzed in SPSS software, version 18, using statistical test Chi-square and central qualitative and distribution statistics. The frequency of lice contamination was 27 cases (4.33%) in total, all of whom were girls. Although the difference between the head lice contamination and gender was statistically significant (P=0.00), the difference between this contamination type and grade of students and their parents’ education and fathers’ occupation was not significance. The highest rate of contamination (6.73%) was, however, observed in fifth graders. All contaminations were seen in girls, which could be due to their longer hair or wearing scarf as compared with boys. Unfavorable health condition and regional hot weather can be effective as well. Therefore, it is essential to provide the students, especially girls, with health training.

## Introduction

One of the most important indicators of developed countries is the level of health and well being of their people.^1^ Protecting children’s health and development includes the growth and development of the country and has been considered as a valuable investment in its economic growth and political stability.^2^ Head lice contamination is one of the most common parasitic contamination in the world that causes serious health problems for many communities, especially for school children.^3^ It may also have psychological and social outcomes. This condition may lead to the students’ educational failure.^4^

Head lice contamination is common worldwide and has been proposed as a major health problem not only in poor countries but also in developed and industrial countries. Every year, over 12 million Americans are contaminated by this parasite.^5^ Studies carried out in different parts of the world have reported different prevalence for head lice in children. For example, the rate of contamination have been estimated to be 16.59% in India,^6^ 13.3% in Yemen^7^ and 8.9% in Belgium.^8^ Various reports have been published on the percentage of contamination in Iran. For instance, it has been reported to be 0.47% in Aran and Bidgol,^4^ 2.2% in Babol,^9^ 4.5% in Gilan,^10^ 6.7% in Hamedan,^3^ 7.7% in Sanandaj,^11^ and 13.3% in Qom.^12^ Considering the importance of students’ health, high prevalence of head lice and its complications among the primary school students, and prevalence of this contamination among rural students of Abadan, we aimed to examine the rate of head lice contamination among the students of primary schools in the villages of Abadan in 2012.

## Patients and Methods

This was a descriptive study on all the primary school students of villages in Abadan performed by a community health nurse in 2012. Cluster random sampling was used in this study. First, the names of all rural health centers, the villages supported by these centers, the primary schools in these villages, the type of school, and the number of students in terms of girls and boys were obtained from the schools’ health center of Abadan College of Medical Sciences. The total number of primary schools in the villages of Abadan was 50 and the total number of students studying in those schools was 2500. Then, 26 schools (14 coeds, 6 female schools, and 6 male schools) and 624 male and female students were randomly selected and examined out of 32 coeds, 10 male schools, and 8 female schools using the formula n=z^2^×p (1-p)/d^2^ in which z=1.96, P=0.5 and d=0.04.^13^ After the introduction given by the researcher and coordination with the School of Medical Sciences of Abadan and other sectors, the necessary permission to cooperate with the researcher was also provided. Entering the selected schools and obtaining permit from the school authorities, the researcher met the participants one by one and personally examined their hair, particularly behind their ears and necks in separate rooms. The results were confidentially recorded in a checklist. With the use of student health records, the researcher filled out the questionnaire for all students. Student’s satisfaction to take part in the study, being a primary school student, and also being originally Iranian while conducting the study were the three inclusion criteria of the study. Therefore, every individual’s agreement on voluntary participation in the study was obtained orally, and the exclusion criteria included lacking one of the three mentioned conditions.

This study was carried out in January and February. Finally, the obtained data were analyzed using SPSS software, version 18, using Chi-square and central qualitative and distribution statistics. 

## Results

Of the total students surveyed, 322 (51.60%) were girls and 302 (48.40%) were boys. Students were in the age range of 6 to 12 years and studied in the first to sixth grades. The frequency of lice contamination was 27 cases (4.33%) in total, all of which were seen in girls (i.e 8.39% of the total girls). Chi-square test showed a statistically significant difference between the head lice contamination and gender (P=0.00). The distribution of head lice contamination in fifth graders was 6.73%, which was higher than other investigated grades. Chi–square test did not show a statistically significant relationship between the head lice contamination and academic grades and age (P=0.75, table 1).

**Table 1:** The Relationship between Head Lice Contamination and Gender and Grade

**Variables**	**Number**	**Infected**	**Number**	**Uninfected**	**Total**	**Percentage**	**P**
		**Percentage**		**Percentage**	**Number**		
Gender							
Girl	27	8.39	295	91.61	322	100	0.00
Boy	0	0	302	100	302	100	
Total	27	4.33	597	95.67	624	100	
Grade							
First	5	4.81	99	95.19	104	100	0.75
Second	3	2.88	101	95.12	104	100	
Third	3	2.88	101	95.12	104	100	
Fourth	5	4.88	99	95.19	104	100	
Fifth	7	6.73	97	93.27	104	100	
Sixth	4	3.85	100	96.15	104	100	
Total	27	4.33	597	95.67	624	100	

Most students contaminated with head lice had parents with elementary education. Their fathers were self employed (79.16% were farmers, ranchers, workers, etc.) and their mothers were housekeepers (100%, table 2). Consistently, most of the parents of healthy students had the same jobs and no statistically significant difference was found between the head lice contamination of students and father’s occupation and parents’ education level.

**Table 2:** The Frequency Distribution of the Contaminated Students Based on Their Parents’ Education and Occupation

**Variables**	**Father**	**Percentage**	**Mother**	**Percentage**
	**Number of Infection**		**Number of Infection**	
Education				
Uneducated	7	25.93	8	29.63
Elementary-middle	19	70.37	19	70.37
Diploma and above	1	3.70	0	0
Occupation				
Governmental	1	3.70	0	0
Self-employed	22	81.48	0	0
Unemployed	4	14.18	27	100

## Discussion

The overall prevalence of head lice contamination among the students of rural primary schools in Abadan was 4.33% and all infestations were seen in girls (i.e 8.39% of the total number of girls). To be specific, the girls were contaminated 8.39 times more than the boys and this difference was significance. 

The findings of this study are consistent with those of a similar study carried out in the city of Aran and Bidgol during 2007-2008 where the amount of head lice contamination in the female and male students of primary schools was 0.42% and 0.05%, respectively.^4^ The findings are also consistent with other studies carried out in Iran where the rate of contamination was reported to be 2.2% in Babol, including 3.48% girls and 0.96% boys;^9^ 4.5% in Gilan, including 5.7% girls and 3.3% boys;^10^ and 4.8% in Khaje, including 6.66% girls and 2% boys.^14^ In the city of Hamedan, the contamination rate was 6.7% and the number of girls who had been contaminated was more than that of boys.^3^ The studies carried out abroad also support the results of this study. According to those studies, this rate reached 16.59% in India, including 20.42% girls and 13.86% boys;^6^ 13.3% in Yemen, including 18.9% girls and 8.6% boys;^7^ and 9.1% in Turkey, including 16.4% girls and 2.1% boys.^15^ As it can be seen, in all of the above mentioned studies, the rate of contamination was higher in girls than boys.

The reasons behind this may be related to the behavioral differences of the two genders, such as short hair of the boys versus long hair of the girls, hair covering with the scarf by the girls, long and intimate contacts among the girls, and finally very short contacts and rough games among the boys.^3^ In this study, there was an increase in the rate of contamination in higher grades which was in concordance with the findings of the studies carried out in Sanandaj^11^ and Qom.^12^ This could be due to the fact that their mothers did not care too much about their health at this age.

The problem of students with the Arabic language was the limitation of this study which made the communication a little difficult but their good cooperation can be regarded as the strength of the study. 

## Conclusion

Since the obtained head lice contamination rate in this study is high, the health condition in the rural primary schools of Abadan is worrying and needs special attention. Therefore, specific training of girls about healthy hair care and prevention of head lice contamination and also its treatment is quite necessary. Monitoring and treating the contaminated students seem to have a higher priority, too. Because lice contamination in students spreads very fast and can consequently infect their family, it is suggested that the parents be examined and provided with necessary training in order to prevent and cure this disease. Health instructors as members of the school’s health group can play an effective role in promoting the health of students. They can provide the students with useful materials using theoretical and practical teaching, pictures, movies, posters, etc. Also, they can recognize the health problems of students through frequent screening and thus help to remove or decrease them or refer the students to the relevant physician; therefore, it would be very helpful to have an educated health instructor in schools. 
